# Synthesis and characterization of highly efficient and recoverable Cu@MCM-41-(2-hydroxy-3-propoxypropyl) metformin mesoporous catalyst and its uses in Ullmann type reactions

**DOI:** 10.1038/s41598-022-08902-w

**Published:** 2022-03-23

**Authors:** Zahra S. Robatjazi, M. Reza Naimi-Jamal, Mahdieh Tajbakhsh

**Affiliations:** Research Laboratory of Green Organic Synthesis and Polymers, Department of Chemistry, Iran University of Science and Technology, P.O. Box 16846-13114, Tehran, Iran

**Keywords:** Catalysis, Organic chemistry

## Abstract

The functionalized MCM-41-(2-hydroxy-3-propoxypropyl) metformin was prepared and anchored by copper ions to employ as a catalyst for the Ullmann C-X coupling reaction. The catalyst was characterized by Fourier-transform infrared spectroscopy, thermogravimetric analysis, X-ray diffraction, transmission electron microscopy, scanning electron microscopy, energy-dispersive X-ray spectroscopy measurements and, N_2_ adsorption–desorption isotherms. The benefits of this catalyst are the use of inexpensive and non-toxic metformin ligand, easy catalyst/product separation, and catalyst recycling. The catalyst can be reused at least for five repeated cycles without a significant loss of its catalytic activity or metal leaching.

## Introduction

Since the first report of periodic mesoporous silica designated as MCM-41 in the early 1990s ordered mesoporous silica materials have attracted much attention^1^. Many new synthetic methods based on heterogenization of homogeneous catalysts have become a major area of research since the potential advantages of these materials such as simplified recovery and reusability over homogeneous systems can have positive environmental effects^2^. Many porous materials have technical applications as heterogeneous catalysts and excellent adsorbents^3,4^. In particular mesoporous compounds, have gained a special place^5,6^. In the last decade, among the different types of mesoporous materials used in heterogeneous catalysis, interesting research was carried out on MCM-41 materials^7–9^, because of some valuable properties, such as larger surface area, high reactivity, good accessibility, and suitable pore structure, chemical, and thermal stability. Much research has been done on the use of these compounds in biomass^10^. for example, Wang et al.^11^ prepared a series of Sn-containing mesoporous MCM-41 catalysts for the conversion of pyruvaldehyde to ethyl lactate. Multicomponent reactions, such as Ugi^12^, Gewald^13^ or coupling reactions^4^, and redox^9,14^ have also been reported using these functionalized MCM-41 catalysts.

Transition metal-catalyzed carbon–oxygen and carbon–sulfur bond formation with aryl halides and aromatic phenols and thiols as electron-pair donors is a powerful tool to prepare O- and S-containing compounds that have high applicability in synthetic, biological, pharmaceutical, and materials science^15^. Many metals such as palladium, nickel, copper, iron, and gold have been able to catalyze this type of reaction^16^. Traditional methods for the synthesis of aryl-sulfur bonds are different and often require harsh reaction conditions^17^. For example, the coupling of copper thiolates with aryl halides requires polar solvents such as HMPA and temperatures around 200 °C. Reduction of aryl sulfones or aryl sulfoxides requires strong reducing agents such as DIBAL-H or LiAlH_4_^18^.

Copper-catalyzed coupling reactions of aryl halides with nucleophiles, so-called Ullmann-type reactions^19^, are well-established methods for preparing pharmaceutically and materially important compounds. Many scientists have reported different conditions for these types of reactions in recent years using copper-based catalysts^20^. This reaction was also carried out in 2014 using thioamides as a source of sulfur^21,22^. In 2021, the Ullmann reaction was used to produce endochin-like quinolone compounds, which are safe treatments for a range of important human and animal afflictions in *Sovitj Pou* research group^23^. Also, cellulose-supported poly(hydroxamic acid) − Cu(II) complex was successfully applied to the Ullmann etherification^24^*.* Various functionalized 2-aminobenzo[*b*]thiophenes have been synthesized at room temperature by the Ullmann coupling reaction in the presence of different Cu salts and 1 10 phenanthroline as ligand^25^. *Ge* research group prepared three different types of functionalized chitosan and anchored with copper salts for use as the catalyst for the Ullmann C–X coupling reaction^26^. Following the interest in Ullmann-type reactions and mesoporous materials, in this work, we report a new recoverable catalyst based on modified MCM-41 anchored with copper ions, which is an efficient catalyst for the Ullmann type reactions.

## Experimental

### Materials

All commercially available chemicals, solvents, reagents and were purchased from Sigma-Aldrich and Merck company, briefly cetyltrimethylammonium bromide (CTAB) (Sigma-Aldrich, ≥ 99%), ammonium hydroxide solution (Sigma-Aldrich, ≥ 99.99%), tetraethyl orthosilicate (TEOS) (ACROS, 98%), EtOH (Sigma-Aldrich, ≥ 99.8%), anhydrous *N, N*-dimethylformamide (Sigma-Aldrich ≥ 99.8%), metformin hydrochloride (Merck, ≥ 99.9%).

The melting points of the prepared derivatives were measured by an Electrothermal 9100 apparatus, which was reported without any correction. The FT-IR spectra were recorded in the range of 400–4000 cm^−1^ using a Shimadzu IR-470 spectrometer by using KBr pellets. ^1^H-NMR spectra were recorded using the Bruker DRX-500 and 300 AVANCE spectrometer. Elemental analysis was provided by EDX analysis, which was recorded by TESCAN4992. The morphology of the synthesized catalyst was studied by SEM using VEGA2 TESCAN instrument. TGA of the prepared catalyst was obtained by an STA504. The XRD measurement of the catalyst was recorded with the X′ Pert Pro diffractometer operating with (40 mA, 40 kV). N_2_ adsorption–desorption isotherms of Cu@MCM-41-HPr-Met nanocomposite were measured at the temperature of liquid nitrogen with a Micromeritics system. The surface area of the nanoparticles was calculated using the Brunauer–Emmett–Teller (BET) method. All products we compared based on their spectra and physical data recorded in the references.

### Catalyst preparation

**Scheme 1:**
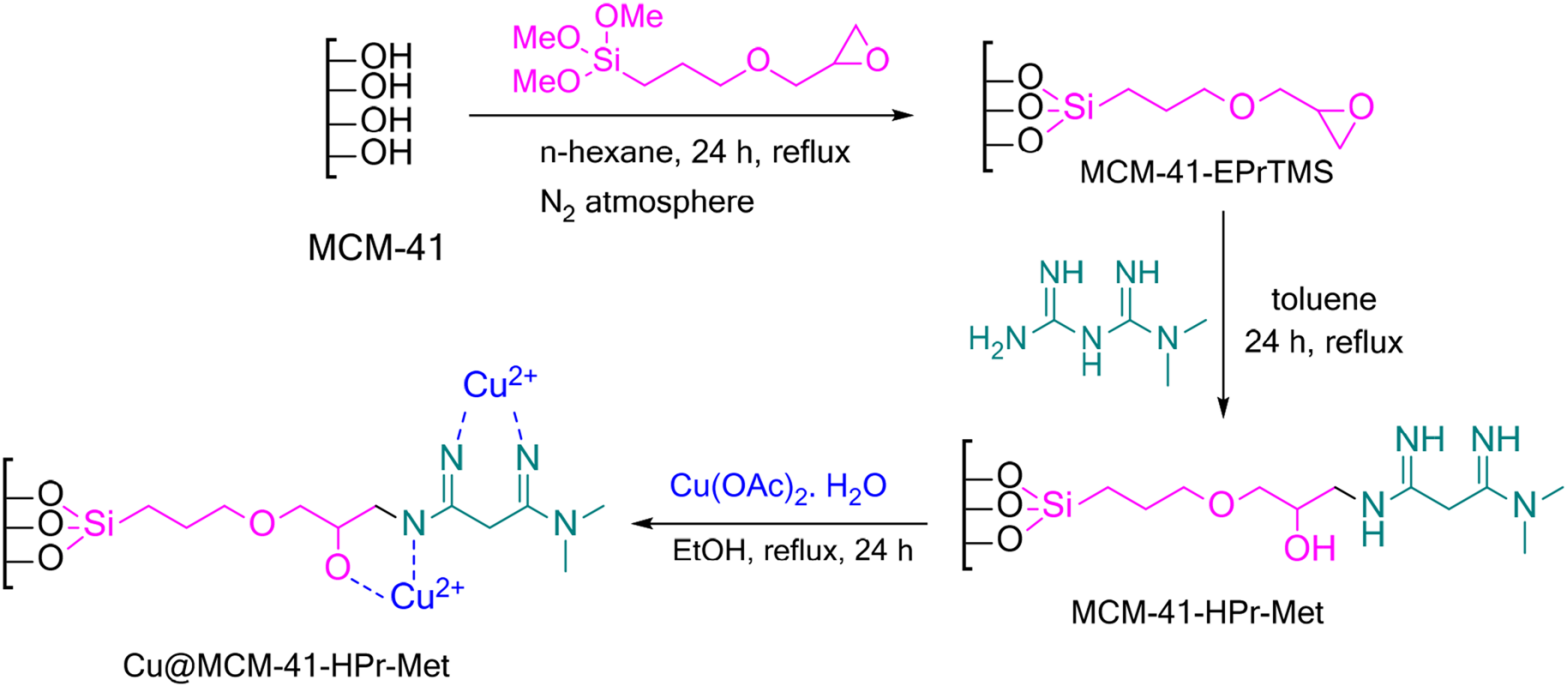
Schematic representation of the synthesis of heterogeneous catalyst Cu@MCM-41-HPr-Met.

The catalyst has been prepared according to Scheme 1, as follows. The given protocol has been used for the preparation of the catalyst on a 5 g scale.

#### Preparation of MCM-41

The MCM-41 synthesis is performed according to the reported procedure by *Zanjanchi*^27^. In brief, 2.7 g ethylamine was added to 42 ml of deionized water and the mixture was stirred at room temperature for 10 min. The amount of 1.47 g of the surfactant cetyltrimethylammonium bromide (CTAB) was gradually added to the above solution under stirring for 30 min. After further stirring for 30 min, a clear solution was obtained. Then, 2.1 g of TEOS solution was added dropwise to the solution. The pH of the reaction mixture was adjusted to 8.5 by the slow addition of the hydrochloric acid solution (1 M) to the mixture. After precipitate formation, slow stirring for 2 h is necessary, and then the precipitate was separated by centrifuge and washed 3 times. The product was dried at 45 °C for 12 h and calcined at 550 °C for 5 h to decompose the surfactant to obtain the white powder. This powder was used as the parent material to prepare the main catalyst.

#### Preparation of MCM-41-EPTMS

In a typical procedure, a round-bottom flask charged with 0.5 g MCM-41 and 10 mL of n-hexane was added, then 0.5 g (2.11 mmol) [3-(2,3-epoxypropoxy)-propyl]-trimethoxysilane added into the reaction mixture. Reaction after stirring for 24 h under inert N_2_ atmosphere and reflux condition in oil bath, was cooled to room temperature. The solid product MCM-41-EPTMS filtered off and was washed twice with n-hexane, dried in an oven at 70 °C for 24 h.

#### Preparation of MCM-41-HPr-Met

The obtained MCM-41-EPTMS (0.4 g) was dispersed in toluene (15 ml) by sonication for 15 min. Triethylamine (2 mmol, 0.202 g) and metformin (1 mmol, 0.129 g) were added to the above solution. Finally, MCM-41-HPr-Met precipitate was obtained after 24 h under reflux condition and washing filtered product with EtOH.

#### Preparation of Cu@MCM-41-HPr-Met

The MCM-41-HPr-Met precipitate (0.25 g) was added in EtOH (25 mL), then copper (II) acetate monohydrate (1 mmol, 0.199 g) was added to the reaction mixture and stirred under N_2_ atmosphere at 70 °C for 20 h. Finally obtained Cu@MCM-41-HPr-Met precipitate was washed with EtOH and dried at 70 °C for 12 h.

### General procedure for diaryl sulfide derivatives catalyzed by Cu@MCM-41-HPr-Met

A mixture of aryl halide (1 mmol), thiophenol (1.2 mmol), K_2_CO_3_ (2 mmol), and 30 mg Cu@MCM-41-HPr-Met as a catalyst in 3 ml DMSO/EtOH (2:1) was stirred for 6 h at 90 °C. The test tube was filled with inert N_2_ gas and sealed. The progress was monitored by TLC, after the reaction was complete, the test tube was cooled to room temperature. First, the EtOAc solvent was added to the reaction mixture to separate catalyst by filtration, the mixture of reaction was poured in distilled water and extracted with EtOAc (3 $\times$ 15 ml). The organic phase was separated with a separatory funnel and the solvent was evaporated with rotary. The final net product was obtained by column chromatography (EtOAc: n-hexane).

### General procedure for diaryl ether derivatives catalyzed by Cu@MCM-41-HPr-Met

A mixture of aryl halide (1 mmol), phenol (1.5 mmol), K_2_CO_3_ (2 mmol), and 50 mg Cu@MCM-41-HPr-Met as a catalyst in 3 ml DMF/EtOH (2:1) was stirred for 6 h at 90 °C under inert N_2_ gas in a sealed tube. The progress was monitored by TLC, after the reaction was complete, the test tube was cooled to room temperature. To separate catalyst by filtration the EtOAc solvent was added to the reaction mixture, after catalyst filtration, the mixture was poured in distilled water and extracted with EtOAc (3 × 15 ml). The organic phase was separated with a separatory funnel and the solvent was evaporated with rotary. The final net product was obtained by column chromatography (EtOAc: n-hexane).

## Result and discussion

In this research, the preparation of Cu@MCM-41-HPr-Met was done as outlined in Scheme 1. The surface of MCM-41 has many hydroxyl groups, which can be functionalized with alkoxysilane reagents. We used [3-(2,3-epoxypropoxy)-propyl]-trimethoxysilane as a valuable linker. Metformin was used then to open the unstable epoxy ring. Catalyst structure can attach to metals because of its NH and OH groups that can chelate the metal cation easily. Copper (II) cation was chosen because it can catalyze the Ullmann Type reactions.

### Characterization of the catalyst

Spectroscopic and analytical techniques FT-IR, TGA, EDX, SEM, TEM, BET, and XRD were used to determine the structural properties of the catalyst Cu@MCM-41-HPr-Met.

#### XRD analysis

**Figure 1:**
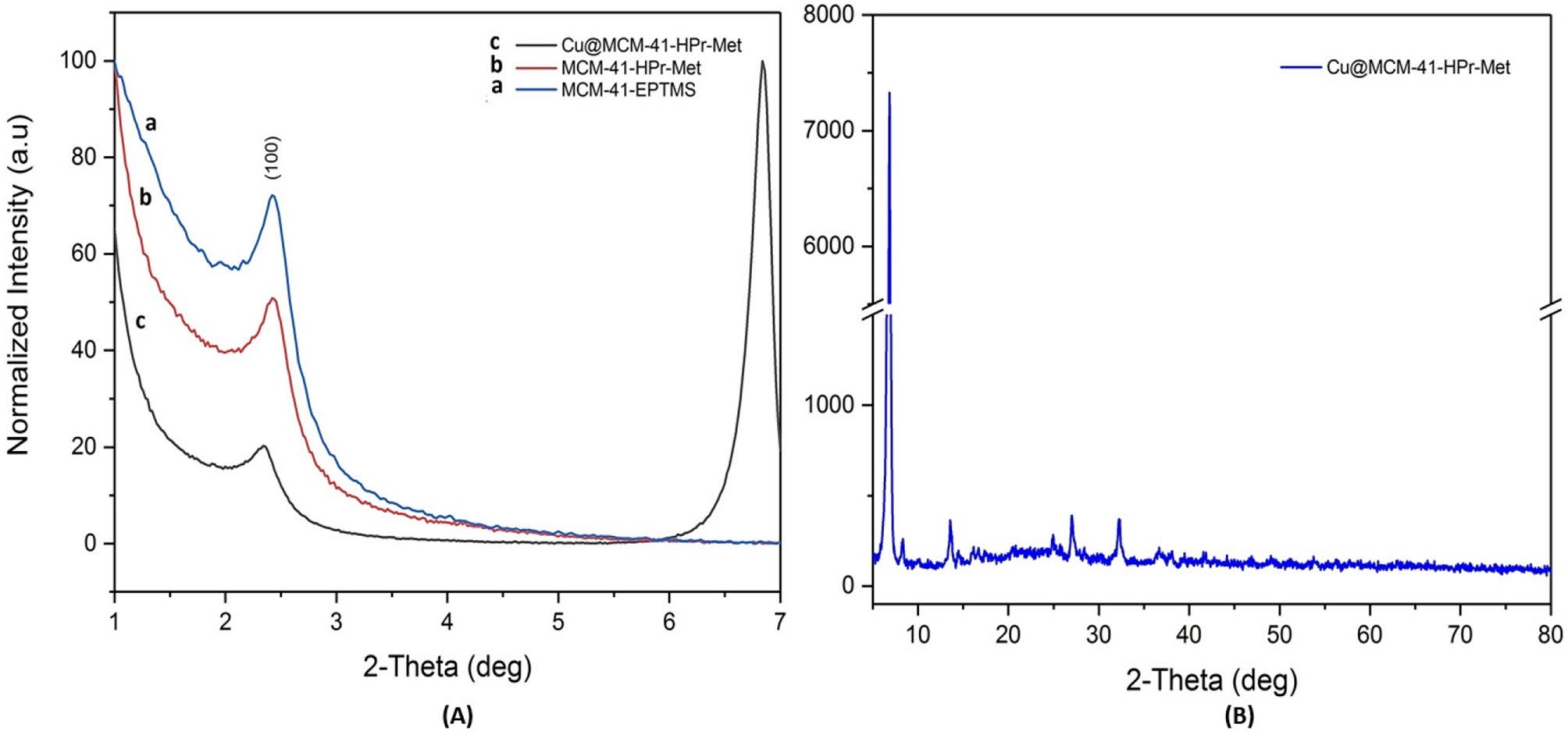
(**A**) The low-angle XRD patterns of a: MCM-41-EPTMS, b: MCM-41-HPr-Met c: Cu@MCM-41-HPr-Met, (**B**) XRD pattern of Cu@MCM-41-HPr-Met.

To identify the crystal structure of the synthesized catalyst, we investigated the low-angle XRD patterns of the modified MCM-41 and Cu@MCM-41-HPr-Met, both were shown in Fig. 1A. The wide-angle XRD pattern of the Cu@MCM-41-HPr-Met was shown in Fig. 1B. The intense 2θ peak at around 2.4° should be attributed to the (100) plane of the MCM-41 and the very weak peak between 4° and 5° was characteristic of its long-range hexagonal structure^28,29^. Comparison of patterns a, b, and c in Fig. 1A shows that the hexagonal structure of the MCM-41 was not destroyed by functionalization, but decreased.

#### FT-IR spectroscopy

**Figure 2:**
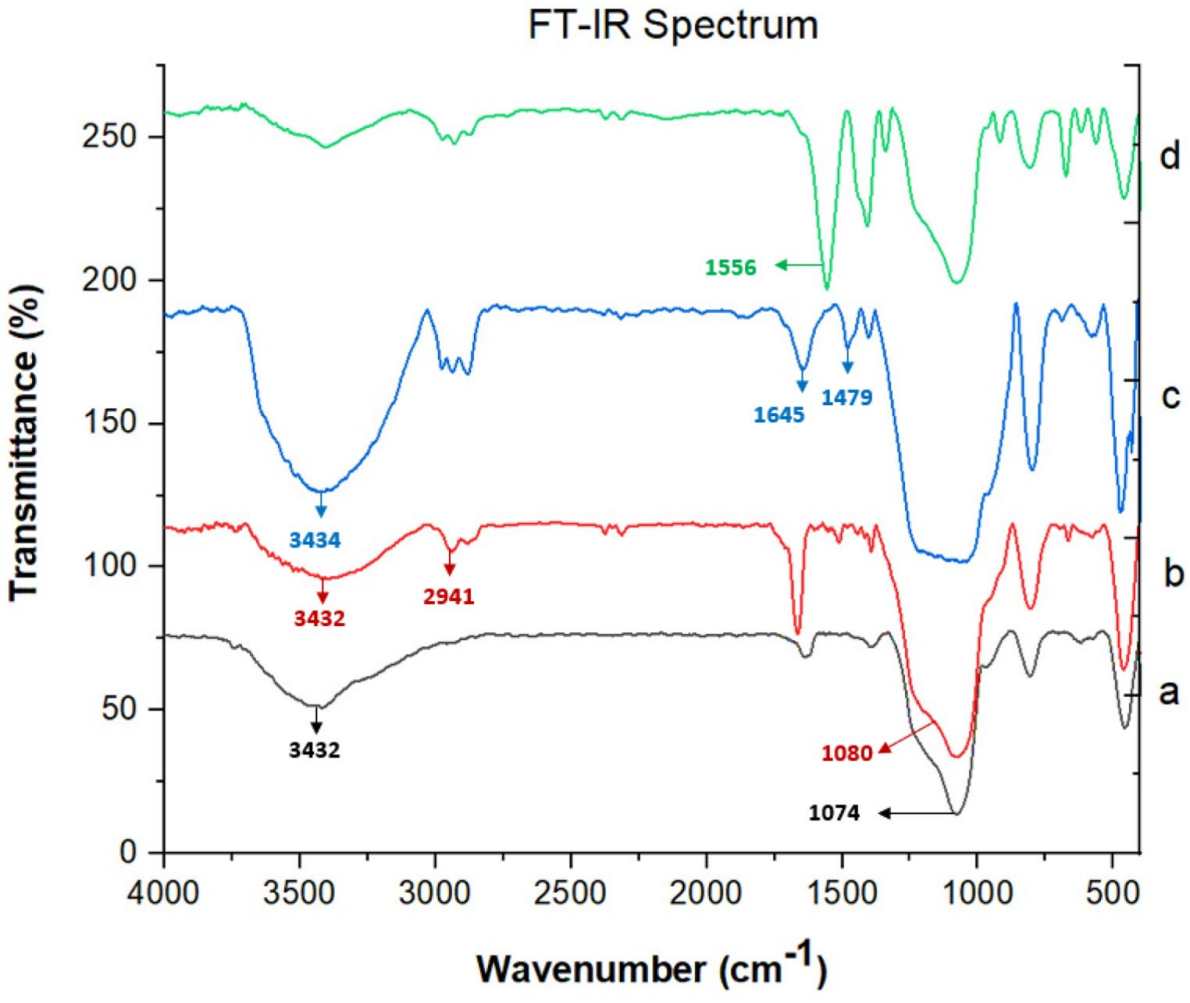
FT-IR spectra of a) MCM-41, b) MCM-41-EPTMS, c) MCM-41-HPr-Met, d) Cu@MCM-41-HPr-Met.

The FT-IR spectra of the synthesis steps are shown in Fig. 2. In spectrum a, the absorption bands at 1074 and 3432 cm^−1^ are related to the stretching vibrations of the O–Si groups, and the OH bond stretching vibrations of MCM-41. In spectrum b, the band observed at 2941 cm^−1^ is attributed to the stretching vibrations of the aliphatic C–H bonds, approving the addition of the propyl group. The wideband observed at 3432 cm^−1^ is due to stretching vibrations of the OH bond. The ether C–O group appears at 1080 cm^−1^. In the c spectrum, the band appearing at 3434 cm^−1^ is attributed to the stretching vibrations of the N–H and O–H groups, and the bands at 1479 and 1645 cm^−1^ represent the C = N imino groups. In the d spectrum, the band shifting to the 1556 cm^−1^, indicates that the copper ion has been successfully doped on the MCM-41-HPr-Met.

#### Scanning electron microscopies (SEM)

**Figure 3:**
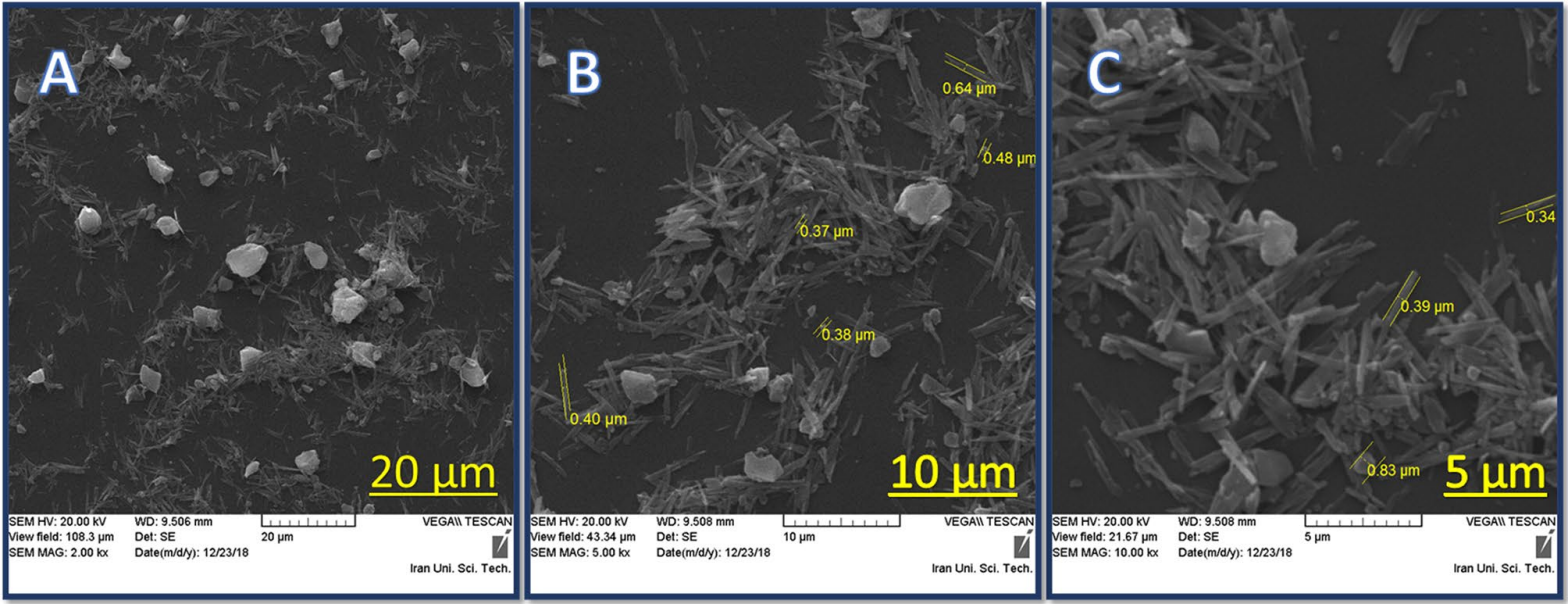
SEM images of Cu@MCM-41-HPr-Met catalyst.

SEM images of the Cu@MCM-41-HPr-Met catalyst are presented in three scales: 5, 10, and 20 µm are shown in Fig. 3. This analysis shows the morphology and size of the synthesized particles. These images show that the particles have spherical morphology as well as a layered structure. As we can see in Fig. 3, we can estimate the particle size between 0.37–0.64 µm.

#### Transmission electron microscopies (TEM)

**Figure 4:**
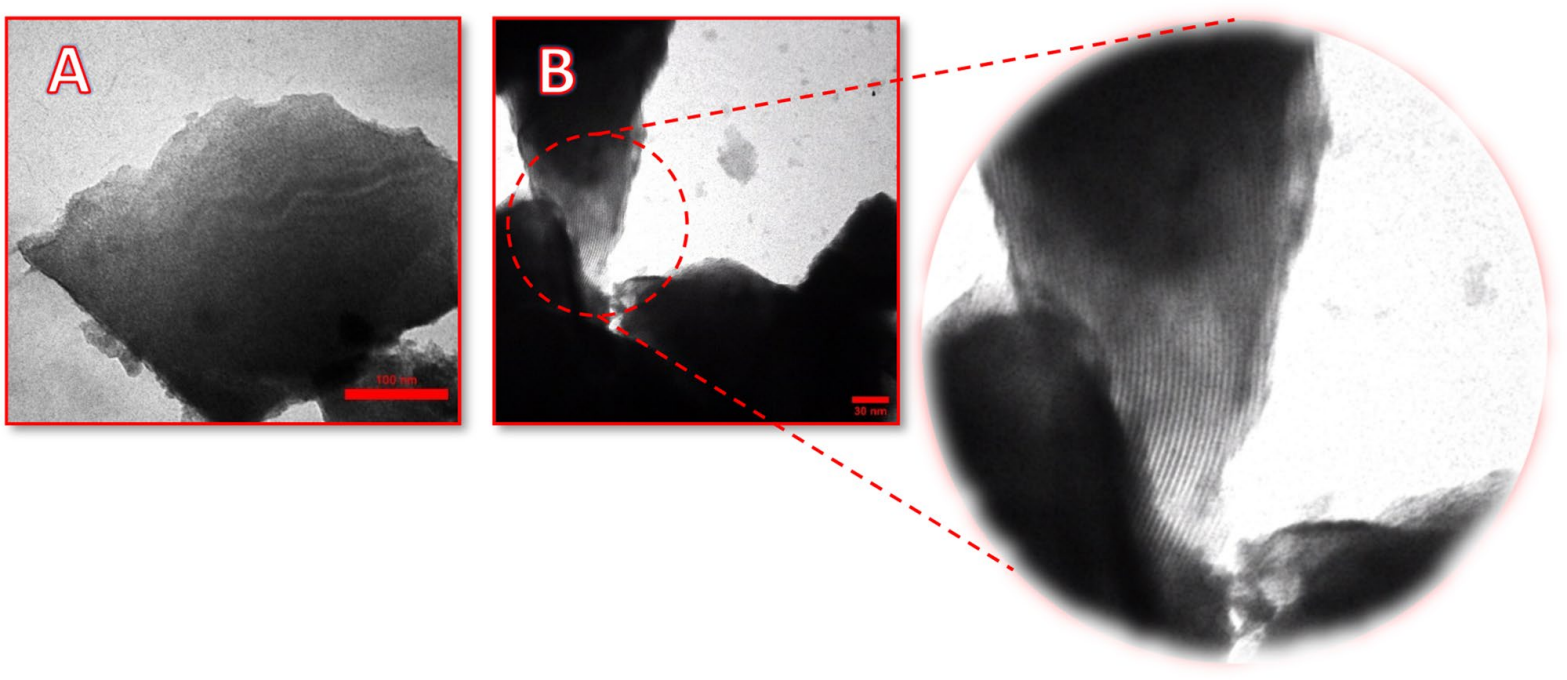
TEM images of Cu@MCM-41-HPr-Met catalyst.

TEM analysis was performed to more accurately study the morphology and particle size of the mesostructured catalyst (Fig. 4A, B). The TEM micrograph in Fig. 4B indicates the ordered mesoporous channels of silica after modification.

#### EDX analysis

**Figure 5:**
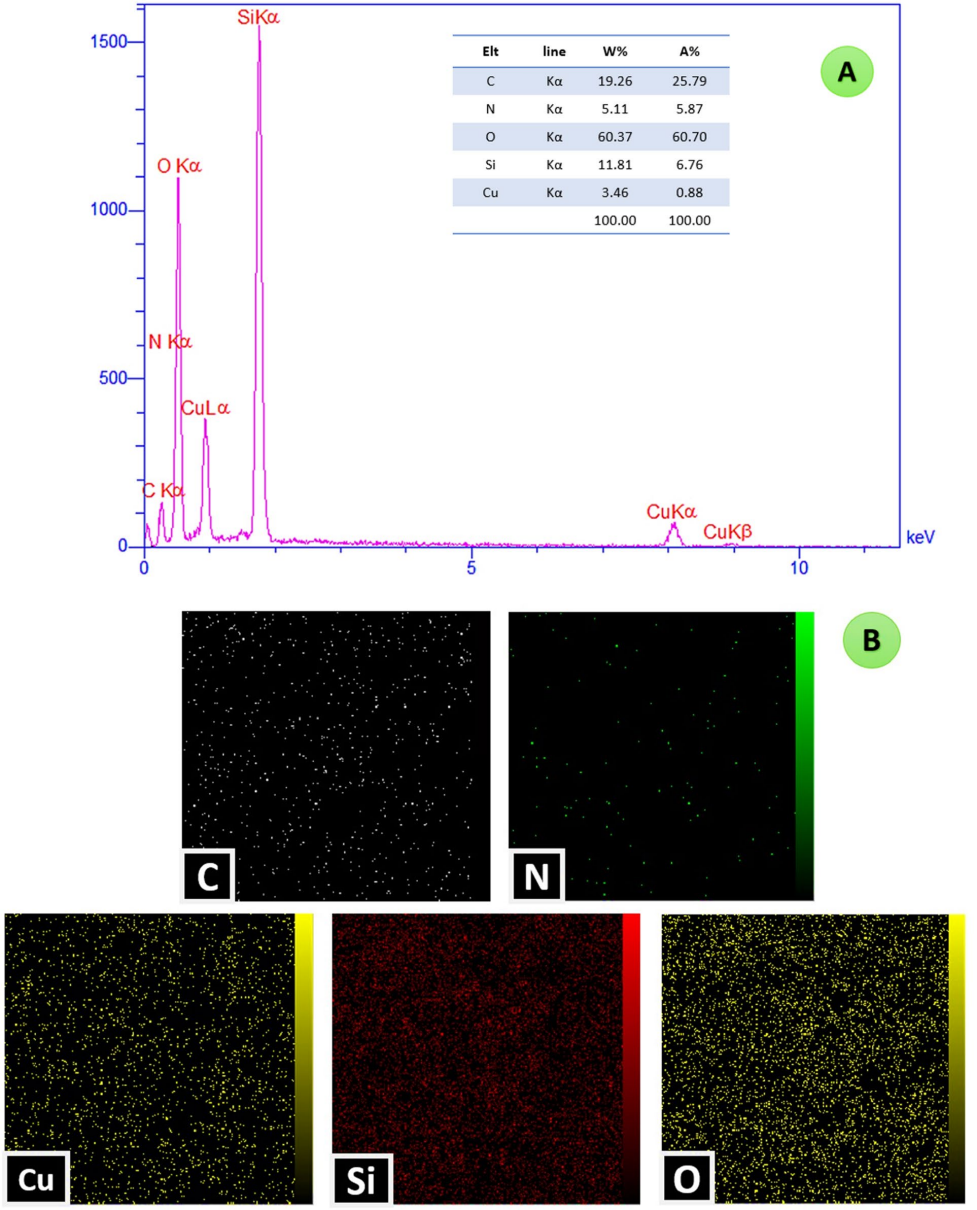
The EDX analysis and mapping images of the Cu@MCM-41-HPr-Met catalyst.

As is shown in Fig. 5A, the presence of expected O, N, Si, C, and Cu elements in the structure of the synthesized nanoparticles were approved by energy dispersive X-ray (EDX) analysis. The presence of the copper ions on the catalyst was confirmed with the bands of 8.04, 8.90 keV (K lines), and 0.92 keV (L line). Moreover, the distribution of the elements in this mesoporous catalyst is shown in the EDX mapping images in Fig. 5B.

#### TGA analysis

**Figure 6:**
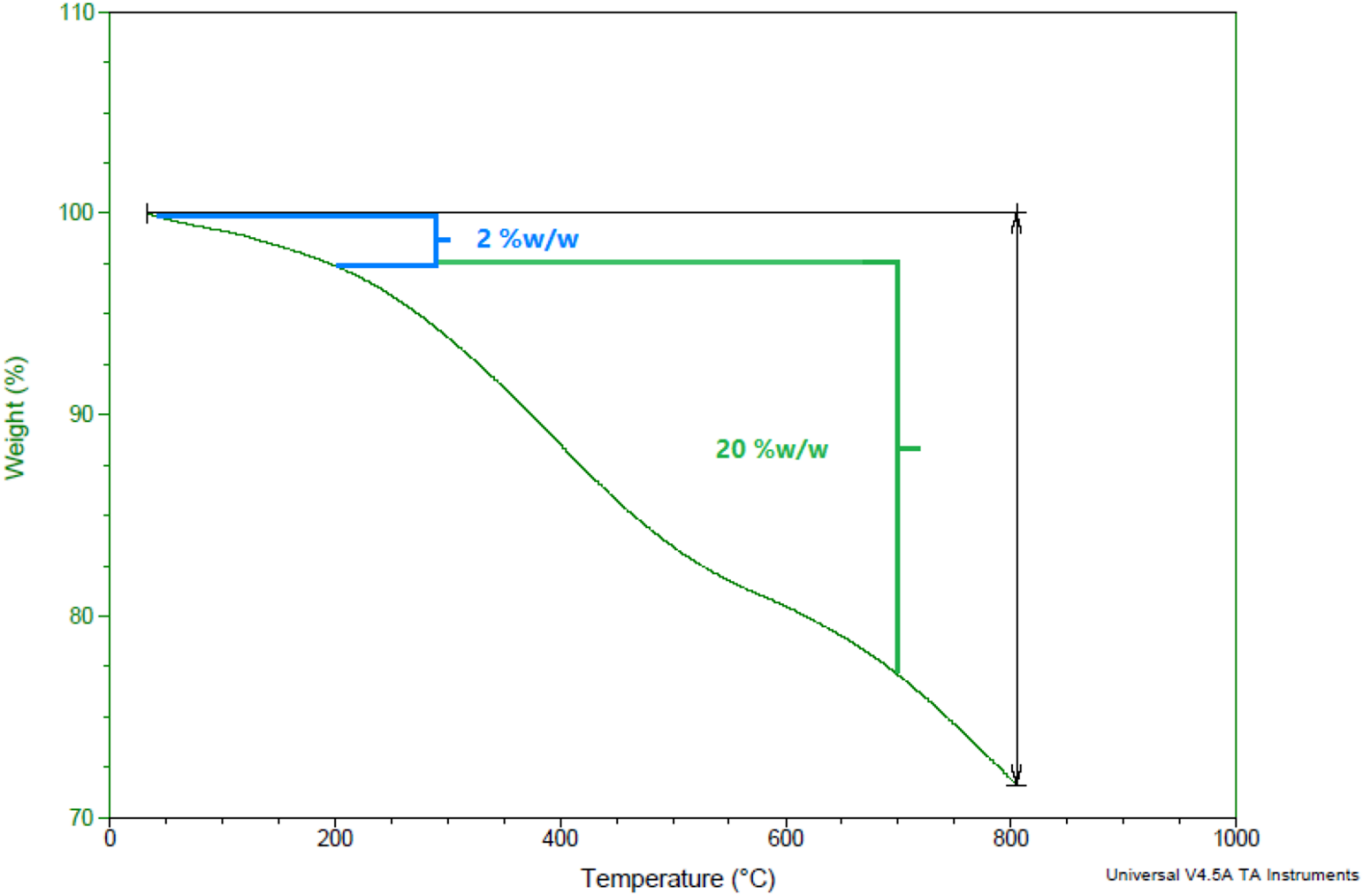
TGA spectrum of Cu@MCM-41-HPr-Met catalyst.

Thermal gravimetric analysis (TGA) can study the behavior of matter versus temperature. In Fig. 6, the downward trend diagram illustrates the fact that the sample mass decreases with increasing temperature. As shown, weight loss below 200 °C corresponds to the removal of the adsorbed water and organic solvents (about 2 wt.%). The second region is mainly related to the thermal decomposition of organic ligands in the temperature range between 200 °C and 700 °C (about 20 wt.%).

#### BET analysis

**Figure 7:**
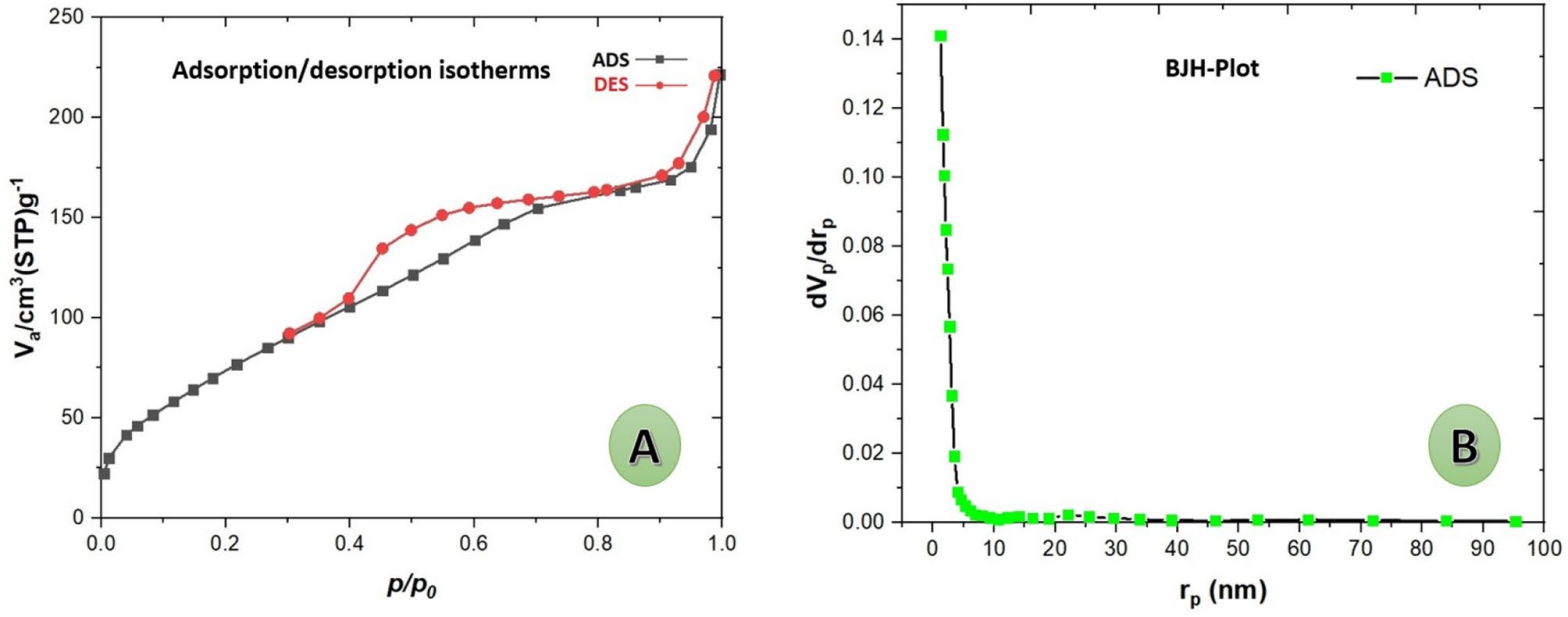
(**A**) Adsorption/desorption N_2_ isotherms and (**B**) pore size distribution of Cu@MCM-41-HPr-Met.

The N_2_ adsorption–desorption isotherm and BJH pore size distribution of the Cu@MCM-41-HPr-Met are shown in Fig. 7A,B. The isotherm is classified as type IV, characteristic of mesoporous materials, with a sharp capillary condensation of nitrogen into the mesoporous channels at high relative pressure and H1 hysteresis loop, which reveals the presence of large channel-like pore structures. Also, based on the shape of its hysteresis, Cu@MCM-41-HPr-Met catalyst has cylindrical pores and the initial structure is retained after functionalization. The structural data of the Cu@MCM-41-HPr-Met catalyst nanoparticles are summarized in Table 1.

**Table 1:** Surface area, pore volume, and pore diameter of Cu@MCM-41-HPr-Met.

Sample	Surface area (m^2^ g^−1^)	Pore volume (cm^3^ g^−1^)	Pore size (nm)
Cu@MCM-41-HPr-Met	301.14	0.3257	4.366

### Application of catalyst in Ullmann type reaction

**Scheme 2:**
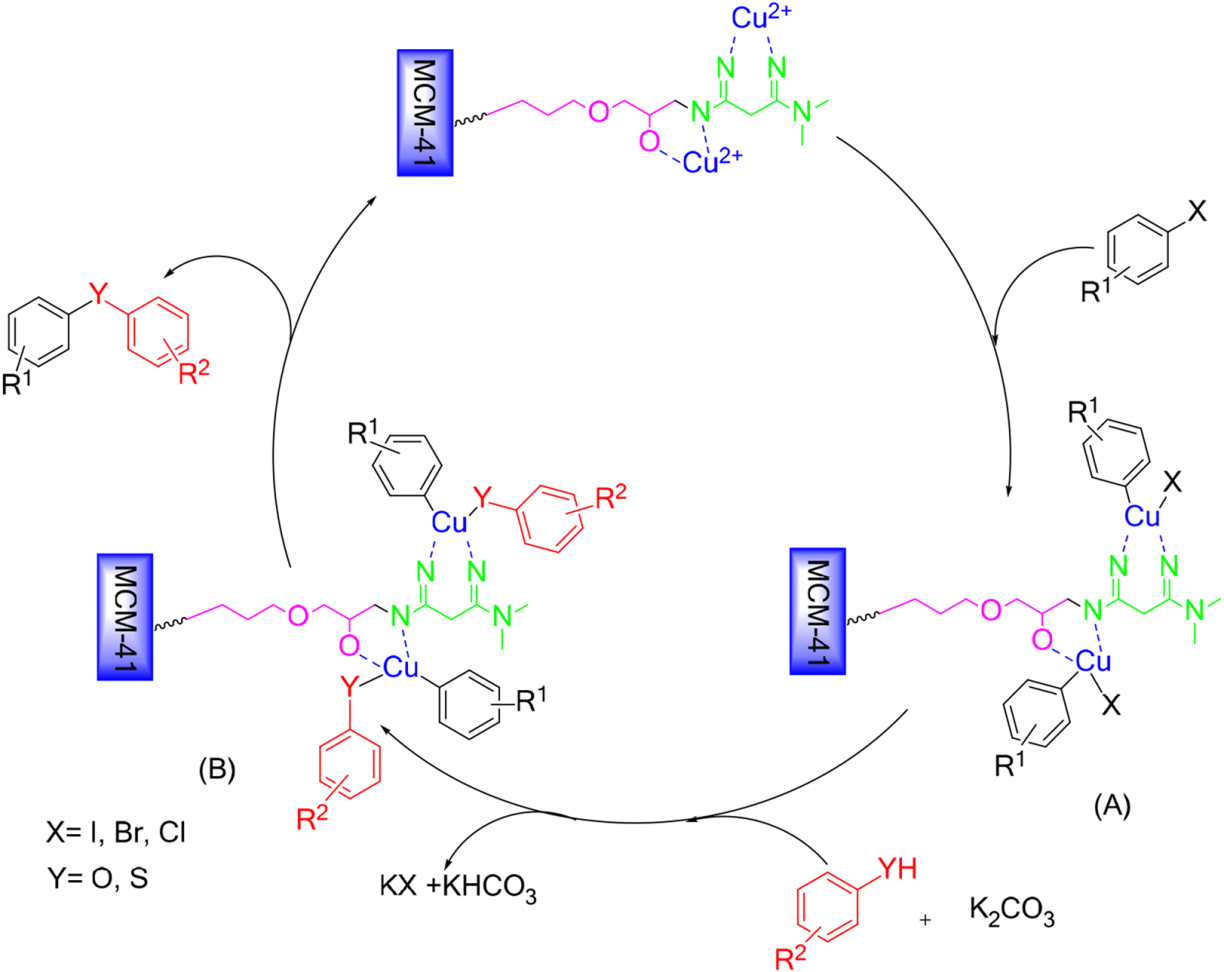
Proposed mechanism for the catalytic activity of Cu@MCM-41-HPr-Met in O-arylation and S-arylation reactions.

We applied MCM-41-HPr-Met to Ullmann-type reactions to show the catalytic utility of the newly constructed structure. We assume that, as shown in Scheme 2, the copper first enters the C-X bond of the aryl halide and produces the intermediate A. (Thio)phenol is converted to its anion in the presence of potassium carbonate as a base, then reacts with the intermediate A and produces intermediate B. When the catalyst leaves, the desired product diaryl sulfides or diaryl ethers are produced.

#### S-Arylation of thiols

To begin with, the reaction between 4-nitro-1-bromobenzene and thiophenol was selected as the model reaction. Then, by optimizing the amount of catalyst and selecting the appropriate solvent and base, accurate time, and temperature measurement, the further reaction progression and product yield increased. As seen in Table 2, by repeating the experiment under different conditions, K_2_CO_3_ was selected as the appropriate base and DMSO/EtOH as a solvent for the reaction. After testing different Cu@MCM-41-HPr-Met catalyst amounts, 30 mg was selected as the optimum amount (Table 3).

**Table 2:** Effect of solvents and different bases in C-S bond formation in a reaction between 1-bromo-4-nitrobenzene and thiophenol as a model reaction.


Entry	Solvent	Base	Catalyst(mg)	Temp. (°C)	Time (h)	Yield (%)
1	Toluene	K_2_CO_3_	30	80	10	67
2	EtOH	K_2_CO_3_	30	80	10	56
3	H_2_O	K_2_CO_3_	30	80	10	0
4	DMSO	K_2_CO_3_	30	80	10	89
5	CH_3_CN	K_2_CO_3_	30	80	10	57
6	DMSO/EtOH	K_2_CO_3_	30	80	10	86
7	Toluene/EtOH	K_2_CO_3_	30	80	10	51
8	CH_3_CN/EtOH	K_2_CO_3_	30	80	10	55
9	DMSO/EtOH	Cs_2_CO_3_	30	80	10	85
10	DMSO/EtOH	KOH	30	80	10	82
11	DMSO/EtOH	NaHCO_3_	30	80	10	80
12	DMSO/EtOH	K_2_CO_3_	30	r.t	10	0
13	DMSO/EtOH	K_2_CO_3_	30	60	10	50
14	DMSO/EtOH	K_2_CO_3_	30	90	10	90
15	DMSO/EtOH	NaOH	30	100	10	90
16	DMSO/EtOH	K_2_CO_3_	30	90	4	81
17	DMSO/EtOH	K_2_CO_3_	30	90	6	95
18	DMSO/EtOH	K_2_CO_3_	30	90	12	88
19^a^	DMSO/EtOH	K_2_CO_3_	–	90	6	0

^a^Without catalyst.

**Table 3:** Optimization of Cu@MCM-41-HPr-Met catalyst in model reaction.

Entry	Solvent	Base	Catalyst (mg)	Temp. (°C)	Time (h)	Yield (%)
1	DMSO/EtOH	K_2_CO_3_	10	90	6	54
2	DMSO/EtOH	K_2_CO_3_	20	90	6	60
3	DMSO/EtOH	K_2_CO_3_	30	90	6	95
4	DMSO/EtOH	K_2_CO_3_	50	90	6	92
5	DMSO/EtOH	K_2_CO_3_	70	90	6	90
6	DMSO/EtOH	K_2_CO_3_	100	90	6	84

After determining the optimal conditions for the reaction of the model, to prove the repeatability of this method and the efficiency of the catalyst, the reaction of derivatives of aryl halides and thiophenols was performed under optimal conditions and diaryl sulfide products were obtained with a high yield. As shown in Table 4, the reactivity with the derivatives of iodobenzene and bromobenzene is higher than that of chlorobenzene. However, studies have shown that doing a reaction with chlorobenzene derivatives with good yield shows Cu@MCM-41-HPr-Met catalyst's high efficacy. In general, the placement of electron-donating groups on aryl halide derivatives increases reactivity, and placing electron-withdrawing groups decreases it.

**Table 4:** Reactions of aryl halides with thiophenols in the presence of Cu@MCM-41-HPr-Met.


Entry	X	R^1^	Thiol (R^2^ =)	Isolated yield (%)	M.P. (°C)
1	I	2-Me	4-Cl	90	111–112
2	I	4-OMe	H	93	29–30
3	I	4-F	4-Cl	93	38–40
4	I	2,4-NO_2_	H	95	121–122
5	Br	4-Me	H	91	91–93
6	Br	4-OMe	H	90	29–30
7	Br	4-Me	Naphthalene-2-thiol	90	70–72
8	Br	4-Me	4-Br	91	77–78
9	Br	2-NO_2_	H	91	75–76
10	Br	2-NO_2_	4-Cl	93	94–96
11	Br	4-NO_2_	H	95	55–57
12	Br	4-NO_2_	4-Cl	94	86–88
13	Br	H	Benzyl thiol	92	50–51
14	Cl	4-Me	H	57	91–93
15	Cl	4-Me	Naphthalene-2-thiol	55	70–72
16	Cl	4-Me	4-Br	52	77–78
17	Cl	2-NO_2_	H	61	75–76
18	Cl	2-NO_2_	4-Cl	60	94–96
19	Cl	4-NO_2_	H	65	55–57
20	Cl	4-NO_2_	4-Cl	62	86–88

Catalyst amount in all reported reactions is 30 mg.

#### O-Arylation of phenols

In this type of reaction, as before, the model reaction was investigated in the presence of DMF/EtOH as the solvent, and K_2_CO_3_ as the base, and reaction time, temperature, and Cu@MCM-41-HPr-Met catalyst amount was optimized (Table 5).

**Table 5:** Optimization of solvents and bases in C-O bond formation in the reaction between Bromobenzene and phenol as a model reaction.


Entry	Solvent	Base	Catalyst (mg)	Temp. (°C)	Time (h)	Yield (%)
1	CH_3_CN	K_2_CO_3_	50	80	10	65
2	H_2_O	K_2_CO_3_	50	80	10	0
3	DMF	K_2_CO_3_	50	80	10	92
4	EtOH	K_2_CO_3_	50	80	10	57
5	CH_3_CN/EtOH	K_2_CO_3_	50	80	10	62
6	DMF/EtOH	K_2_CO_3_	50	80	10	84
7	DMF/EtOH	KOH	50	80	10	60
8	DMF/EtOH	Cs_2_CO_3_	50	80	10	84
9	DMF/EtOH	K_3_PO_4_	50	80	10	81
10	DMF/EtOH	KOH	50	r.t	10	0
11	DMF/EtOH	K_2_CO_3_	50	60	10	45
12	DMF/EtOH	K_2_CO_3_	50	90	10	93
13	DMF/EtOH	K_2_CO_3_	50	100	10	93
14	DMF/EtOH	K_2_CO_3_	50	90	4	85
15	DMF/EtOH	K_2_CO_3_	50	90	6	93
16	DMF/EtOH	K_2_CO_3_	50	90	12	82
17	DMF/EtOH	K_2_CO_3_	–	90	6	0

The reactivity of aryl halides with electron-withdrawing groups in the para position is better than aryl halides with the electron-withdrawing group in the ortho position. Also, the order of reactivity of aryl halides is that aryl bromides are more reactive than aryl chlorides (Table 6).

**Table 6:** The reaction of aryl halides with phenols in the presence of Cu@MCM-41-HPr-Met as a catalyst.


Entry	X	R^1^	Isolated yield (%)	M.P. (°C)
1	Br	H	93	–
2	Br	2-NO_2_	94	52–53
3	Br	4-NO_2_	95	52–53
4	Br	4-CN	91	45–46
5	Br	4-OAc	90	49–50
6	Br	1-Bromonaphthalene	86	51–52
7	Br	2-Bromonaphtalene	88	48–49
8	Cl	2-NO_2_	62	52–53

To show the capability and efficiency of this method and the Cu@MCM-41-HPr-Met as a catalyst, a comparison has been summarized in Table 7 with the previous methods of synthesis diaryl sulfides and diaryl ethers reported in some literature.

**Table 7:** Comparison of the results obtained in the synthesis of diaryl sulfides (1–5) and diaryl ethers (6–10) in the presence of Cu@MCM-41-HPr-Met and other catalysts.

Entry	Catalyst	Conditions and amount of catalyst	Time (h)	Yield (%)	References
1	CuO@GO	DMSO, 110 °C, TEA, 6 mg	14	79	^30^
2	FMNPs@Cu-TPy	DMF, 110 °C, K_2_CO_3_, 80 mg	7	85	^31^
3	CuI/L	Dioxane, 120 °C, Cs_2_CO_3_, 10 mol%	17	90	^32^
4	CuFe_2_O_4_	Dioxane, reflux, *t*-BuOK, 10 mol%	24	62	^33^
5	Cu@MCM-41-HPr-Met	DMSO/EtOH, 90 °C, K_2_CO_3_, 30 mg	6	95	This work
6	Pd/ZnO nanoparticles	DMF, 120 C, K_2_CO_3_, 5 mg	15	80	^34^
7	MWCNTs-Met/CuI	DMF, 80 °C, K_2_CO_3_, 20 mg	12	90	^35^
8	Cu/RGO–Fe_3_O_4_ Nanocomposite	DMF, 120 °C, Cs_2_CO_3_, 50 mg	12	96	^36^
9	CuO–Fe_3_O_4_	DMF, 145 °C, Cs_2_CO_3_, 2 mmol	24	89	^37^
10	Cu@MCM-41-HPr-Met	DMF/EtOH, 90 °C, K_2_CO_3_, 50 mg	6	93	This work

### Reusability of the catalyst

**Figure 8:**
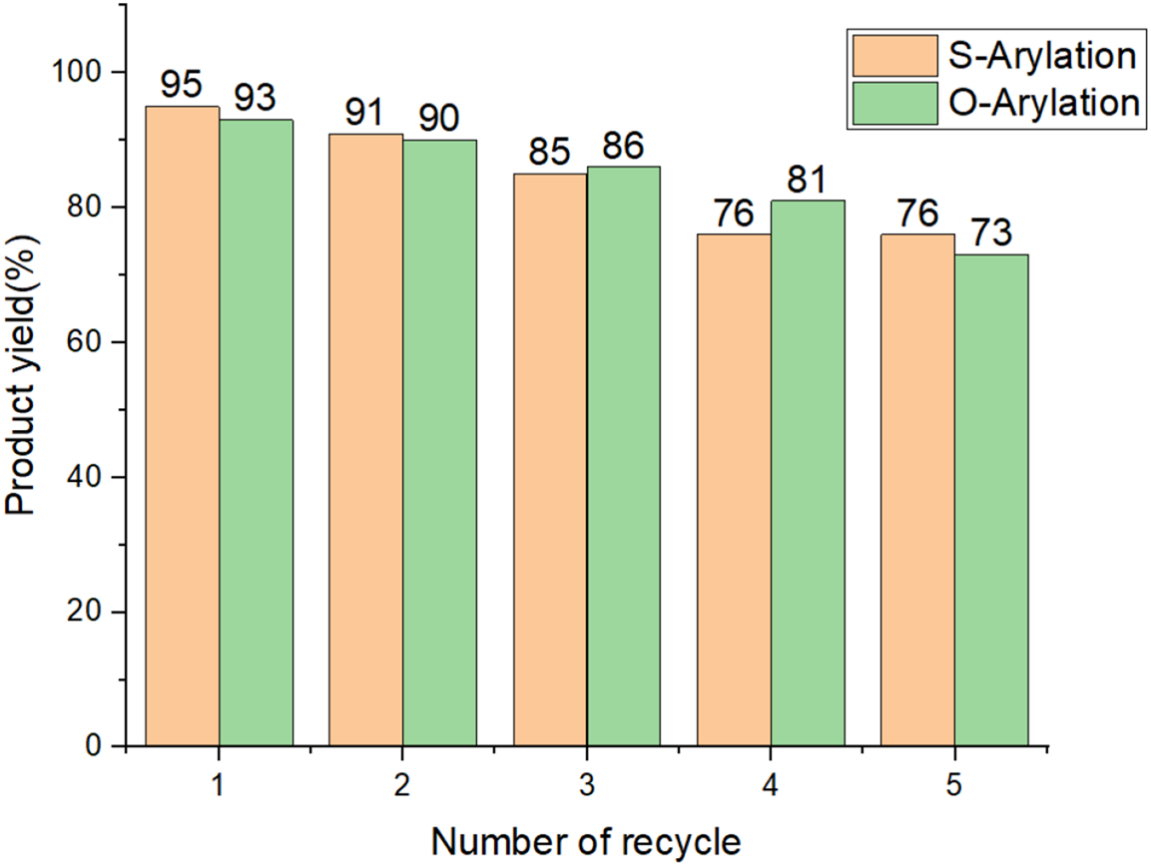
The number of Cu@MCM-41-HPr-Met catalyst recovery in the synthesis of diaryl sulfides and diaryl ether.

One of the most important issues with heterogeneous catalysts is their effective lifespan and their ability to be recycled and reused. Therefore, this was also examined in the present catalytic system. For this purpose, after the reaction was complete, the Cu@MCM-41-HPr-Met catalyst was separated using a strainer and washed several times using ethyl acetate. The Cu@MCM-41-HPr-Met catalyst was then placed in an oven to dry. We used the catalyst again in the (thio)phenol's reaction with 1-bromo-4-nitrobenzene as a model reaction. This operation was repeated 5 times and they give the results for both types of reactions in Fig. 8. As you can see, there were no significant changes in the efficiency or activity of the catalyst after repeated use.

## Conclusion

In this paper, we were able to synthesize a new functionalized catalyst based on mesoporous silicates and investigate its reactivity in C–S and C–O bond formation. Considering the advantages of heterogeneous catalysts, Cu@MCM-41-HPr-Met catalyst has such as ease of use, easy separation of products, adaptability to the environment, mild reaction conditions, and most importantly, recyclability of the catalyst.
